# Deciphering the Anode-Enhanced Azo Dye Degradation in Anaerobic Baffled Reactors Integrating With Microbial Fuel Cells

**DOI:** 10.3389/fmicb.2018.02117

**Published:** 2018-09-06

**Authors:** Yonggang Yang, Ou Luo, Guannan Kong, Bin Wang, Xiaojing Li, Enze Li, Jianjun Li, Feifei Liu, Meiying Xu

**Affiliations:** ^1^Guangdong Provincial Key Laboratory of Microbial Culture Collection and Application, Guangdong Institute of Microbiology, Guangzhou, China; ^2^State Key Laboratory of Applied Microbiology Southern China, Guangzhou, China

**Keywords:** microbial fuel cells, anode respiration, azo dye, wastewater treatment, anaerobic baffled reactor

## Abstract

Microbial anode respiration in microbial fuel cells (MFCs) can enhance the degradations of many electron acceptor-type contaminants which are presumed to be competitive to anode respiration. The mechanisms underlying those counterintuitive processes are important for MFCs application but are unclear. This study integrated MFCs with anaerobic baffled reactor (ABR), termed MFC-ABR, to enhance the reduction of azo dye acid orange-7 (AO-7). Compare with ABR, MFC-ABR enhanced the degradation of AO-7, especially at high AO-7 concentration (800 mg/L). Acute toxicity test suggested a higher detoxication efficiency in MFC-ABR. Higher microbial viability, dehydrogenase activity and larger sludge granule size were also observed in MFC-ABR. MFC-ABR significantly enriched and reshaped the microbial communities relative to ABR. Bacteria with respiratory versatility, e.g., *Pseudomonas*, *Geobacter*, and *Shewanella*, were significantly enriched. Functional prediction showed that six metabolism functions (manganese-, iron-, fumarate- and nitrate-respiration, oil bioremediation and chemoheterotrophy) were significantly stimulated while methanogenesis, sulfate-respiration, hydrogen-oxidation were suppressed in MFC-ABR relative to ABR. The results provided important information for understanding the role of microbial anode respiration in contaminated environments.

## Introduction

Azo dyes are the most extensively used synthetic dyes in various commercial applications and 10–20% of them are lost in effluent during dyeing process (Li et al., 2010; Solis et al., 2012; Solanki et al., 2013). Those commercial dyes are generally highly stable in light and resistant to aerobic biodegradation. Wastewaters containing azo dyes or their breakdown products are usually toxic, mutagenic and inhibit light penetration in water bodies, hence affecting aquatic ecosystems (Mu et al., 2009; Sun et al., 2009). Toxication events caused by azo dye containing effluents are increasingly reported (de Aragao Umbuzeiro et al., 2005).

Biodegradation is a widely used methods for azo dye treatment in which the microbial azo reduction under anaerobic condition was considered to be the rate-limiting step (Solis et al., 2012). Many efforts have been made to stimulate the anaerobic azo reduction. Microbial fuel cells (MFCs) is a group of bioelectrochemical system (BES) capable of converting the contaminants in wastewater into electricity via microbial extracellular electron transfer (EET) to anode (Solanki et al., 2013; Jiang et al., 2018; Zhao et al., 2018). It has been shown that azo reduction could be enhanced by either anode or cathode in MFCs (Mu et al., 2009; Sun et al., 2009; Solanki et al., 2013). Moreover, by integrating BESs with traditional wastewater treatment reactors, the treatment efficiency and feasibility could be further enhanced compared to the traditional reactors or BESs (Cui et al., 2014; Kong et al., 2015; Xu et al., 2018a).

The cathode-enhanced azo dye reduction in an MFC is expectable as azo dyes can serve as electron acceptors to be reduced electrochemically or biologically by cathode or cathodic biofilms (Mu et al., 2009). However, several reports have shown anode-stimulated azo dye reduction which were counterintuitive as anode, as another electron acceptor for microbes, was assumed to be competitive to microbial azo dye reduction (Sun et al., 2009; Liu et al., 2013; Solanki et al., 2013). In addition to azo dyes, the biodegradation of some other electron acceptor-type contaminants [e.g., nitrophenol, chlorinated hydrocarbons and vanadium (V)] could also be stimulated by MFC anodes (Pham et al., 2009; Hao et al., 2016; Jiang et al., 2018; Xu et al., 2018b; Zhao et al., 2018). Electron acceptors such as oxygen, anode, nitrate, Fe(III) are usually used to stimulate biodegradations in wastewater treatment or bioremediation. Therefore understanding the anode-enhanced biodegradation of azo dye will be important to MFC application and other electron acceptor-driven biostimulation processes. However, the mechanism underlying such counterintuitive phenomenon is still unclear.

In this study, we integrated MFC electrodes with an anaerobic baffled reactor (ABR), a traditional wastewater treatment reactor to enhance the degradation of a commercial sulfonated azo dye Acid Orange 7 (AO-7). The AO-7 degradation, COD removal and effluent toxicity were evaluated. Moreover, the sludge particle size, microbial viability and dehydrogenase (DHA) activity, microbial community structure and function were comparatively analyzed to decipher the anode-enhanced azo dye degradation in MFC-ABR.

## Materials and Methods

### Anaerobic Baffled Reactors Coupling With Microbial Fuel Cells (MFC-ABR)

Each MFC-ABR and ABR reactors were made of plexiglass [353 mm (length) × 80 mm (width) × 400 mm (height)] with an effective volume of 8.4 L (**Supplementary Figure S1**). Each reactor contained three chambers which were separated by two vertical over-flow plates. Each compartment is further divided into an up-flow and a down-flow part by a vertical baffle, the volume of the two part is 4:1. The heights of the over-flow plates decreased from 40 to 35 and 30 cm to allow the influent flows from one compartment to the next. The bottom of each baffle plates have a 45° slant edge and a distance of 1 cm to the ABR inner bottom.

The anode of the MFC module is carbon brush that made by bonding plain carbon fibers (Liso Composite Material, China) to a stainless steel wire (15 cm in length and 5 cm in diameter), while the cathode is a piece of rectangle carbon felt (5 × 4 × 1 cm, Liso Composite Material, China). Both anodes and cathodes were used after being boiled in deionized water for 20 min. The anode was embedded in the sludge in each chamber and the carbon felt cathode was fixed at the water surface.

Six reactors were assembled and contained an anode and a cathode in each chamber. The anodes and cathodes of three reactors was connected by titanium wires with 1000 ohm resistors, i.e., MFC-ABR. The cathodes and anodes in the other three reactors was not connected, i.e., ABR.

### Inoculation and Operational Conditions

The ABR and MFC-ABR was inoculated with sludge from a secondary settling tank at Liede wastewater plant (Guangzhou, China). Before inoculation, the sludge was stored in a bucket at room temperature (23 ± 3°C) for 1 day. Then, the overlying water was removed and the sludge was added into the ABR and MFC-ABR reactors. Each chamber was inoculated with 1.1 L of sludge to embed the anode.

Medium for all reactors contained (per Liter): 0.41 g CH_3_COONa, 0.407 g NH_4_Cl, 6 g Na_2_HPO_4_, 3 g KH_2_PO_4_, 0.5 g NaCl, 0.1 g MgSO_4_⋅7H_2_O, 0.015 g CaCl_2_⋅7H_2_O. And the concentration of AO-7 was increased from 200 (stage I, 27 days) to 400 (stage II, 28 days) and 800 (stage III, 25 days) mg/L to evaluate the performances of ABR and MFC-ABR under different azo dye concentrations. The medium was pumped into all reactors with peristaltic pumps with a hydraulic detention time of 24 h. The experiments were conducted at ambient temperature.

### Chemical Analyses

The effluent for chemical analyses were sampled every 3 days. The samples were centrifuged with 8000 rpm for 5 min, then filtered through 0.45 μm-pore-size syringe filter. AO-7 concentration of the effluent was measured with an UV-visible spectrophotometer (UV-2600, SHIMADZU) at 484 nm. The reduced products of AO-7 degradation were identified and quantified with high performance liquid chromatography (HPLC). The HPLC system used a Zorbax SB-C18 (150 mm × 4.6 mm × 5 μm, Agilent) for separation and a UV detector for measurement at 254 nm (Thung et al., 2018). COD in the effluents was pretreated with a HACH fast digester (HACH 2800) and measured at 600 nm using an UV-visible detector. The particle diameter of the sludge was analyzed using a particle size and shape analyzer (Ankersmid Eyetech) with an analyzing range of 0.1 – 3600 μm. The Coulombic efficiencies of each chamber in different stages were calculated as previously reported (Logan et al., 2006).

### Effluent Eco-Toxicity Test

Acute eco-toxicity of the effluents was tested as reported before (Yin et al., 2005). Briefly, 30 mL of effluent samples were collected from the three chambers of each reactor at the end of the experiments. Prior to measurement, samples were centrifuged at 4000 rpm for 5 min. The supernatant (20 ml) was added into a beaker that contains eight *Daphnia magna* neonates born within 24 h. The number of living neonates was recorded after 24 h. The survival rate of the *Daphnia magna* was used to evaluate the acute toxicity of the effluents. Three replicates were done for each effluent samples.

### Biomass and Microbial Activity Analyses

The sludge and anodes were sampled from each reactor at the end of experiment. The biofilm-covered anode brushes were rinsed in sterilized phosphate buffer (pH = 7.0) to remove loosely attached bacteria cells and sludge residues. The protein-based biomass of the sludge was analyzed by a Bradford-protein quantification kit (PA102, TIANGEN). DHA activity was tested by evaluating the 2, 3, 5 - triphenyl tetrazoliumchloride (TTC) reducing capacity of the sludge samples at a wavelength of 485 nm by a UV-visible spectrophotometer (UV-2600, SHIMADZU) (Bozich et al., 2017). The microbial metabolism activity in sludge and anode biofilms were analyzed using a confocal laser scanning microscopy (CLSM, LSM 700) after being stained by LIVE/DEAD BacLight staining kit (Molecular Probes, Invitrogen), as described in our previous report (Yang et al., 2015). The specific viability (r) of the sludge and biofilms was presented as the ratio of viable to total biofilm cells based on pixel counting (Yang et al., 2015).

### DNA Sequencing and Community Analyses

After stage-III, the sludge samples in different chambers were collected. 10 ml of each sludge sample were centrifuged at 5000 rpm for10 min. DNA of the collected precipitates were was extracted using a PowerSoil^®^ DNA Isolation Kit for Soil (Mo Bio Laboratories Inc.). Amplification of the 16S rRNA gene V4 region was done using the primers of 515F (5′-GTGCCAGCMGCCGCGGTAA-3′) and 806R (5′-GGACTACHVGGGTWTCTAAT-3′) with Illumina adapters. The DNA amplification, sequence processing and statistical analysis were conducted as described in our previous reports (Xu et al., 2014; Sun et al., 2017). The NCBI accession number to the sequence data is PRJNA479585. Before analyses, the sequence data were processed using QIIME Pipeline-Version 1.7.0. All sequence reads were trimmed and assigned to each sample based on their barcodes. Sequences < 200 bp were removed with an average base quality score Q < 25. The 16S rRNA gene sequences were used for chimera check using the Uchime algorithm. Sequences were clustered into operational taxonomic units (OTUs) using a 97% identity threshold. Each sample was rarefied to the sample exhibiting the lowest number of reads for both alpha-diversity (observed species, Shannon’s and Simpson’s diversity index) and beta diversity (PCoA, UniFrac) analyses, for which the rarefaction curves were generated from the observed species. The principal co-ordinates analysis (PCoA) based on the Bray-Curties distance were executed to investigate the relationships among all samples. The redundancy analyses (RDA) were applied to explore the relationships between the samples and environmental variables including pH, dissolved oxygen (DO), oxidation-reduction potential (ORP), voltage and AO-7 removal efficiency. Both of the PCoA and RDA were executed by applying the vegan packages in R software. Taxonomy was assigned using the Ribosomal Database Project classifier at a confidence-level of 80%. OTUs with the abundances exceeding 1% were selected to compare the bacterial communities from different samples unless stated otherwise. FAPROTAX database was used to evaluate the functional composition of the OTUs in the sludge from different reactors (Louca et al., 2016).

## Results and Discussion

### Electricity Generation in MFC-ABR

**FIGURE 1:**
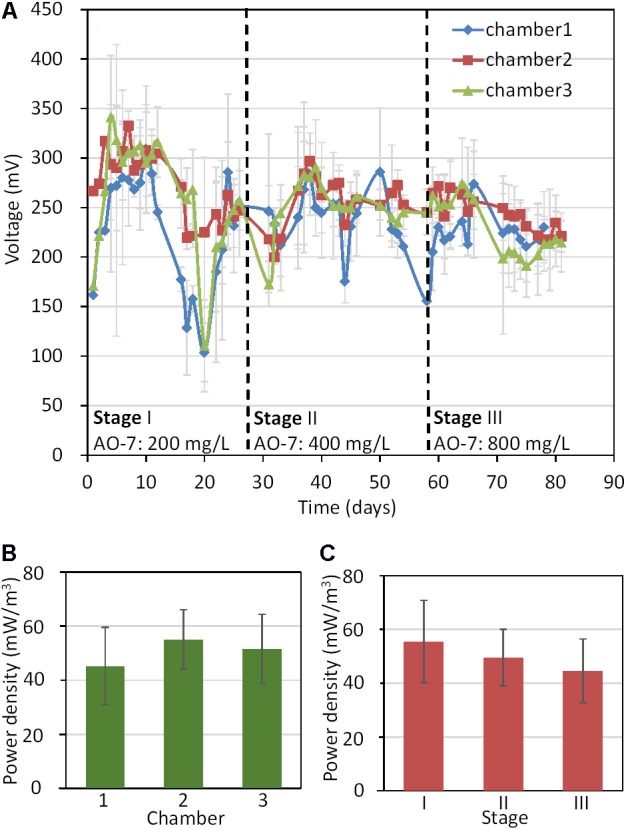
Electricity generation in MFC-ABRs. **(A)** Voltage curve of the three chambers with different AO-7 concentrations; **(B)** The average power densities in different chambers; **(C)** The average power densities in different stages.

Electricity generation in all MFC-ABR chambers increased rapidly after inoculation and reached their maximum voltages within 5 days (**Figure 1A**). Compared with many other MFCs inoculate with anaerobic sludge, MFCs for azo-dyes degradation generally showed more rapid electricity generation (Wang et al., 2009; Gao et al., 2014; Kong et al., 2015) as azo dye reduction products such as aminophenol, phenazine and naphthol can play as electron mediators in electricity generation (van der Zee et al., 2008; Chen et al., 2013). The naphthol generated in AO-7 reduction could be used as an electron mediator by microbes (Chen et al., 2013). The electricity generation of chamber-1 showed the largest variation throughout the experiment probably due to the unstable environments including the dissolved oxygen, pH and temperature caused by the influents. Moreover, chamber-1 showed the lowest average power density (43.5 ± 12.7 mW/m^3^) while chamber II generated the highest (51.6 ± 7.2 mW/m^3^, **Figure 1B**). On the other hand, stage I generated the highest average power density while stage III generated the lowest (**Figure 1C**). These results showed that electricity generation decreased with the increase in AO-7 concentration, suggesting that AO-7 could be used as a more favorable electron acceptor by microorganisms and inhibited electrode reduction in sludge (Yang et al., 2015; Thung et al., 2018).

### AO-7 Degradation and Detoxication

#### The Enhanced AO-7 Degradation in MFC-ABRs

**FIGURE 2:**
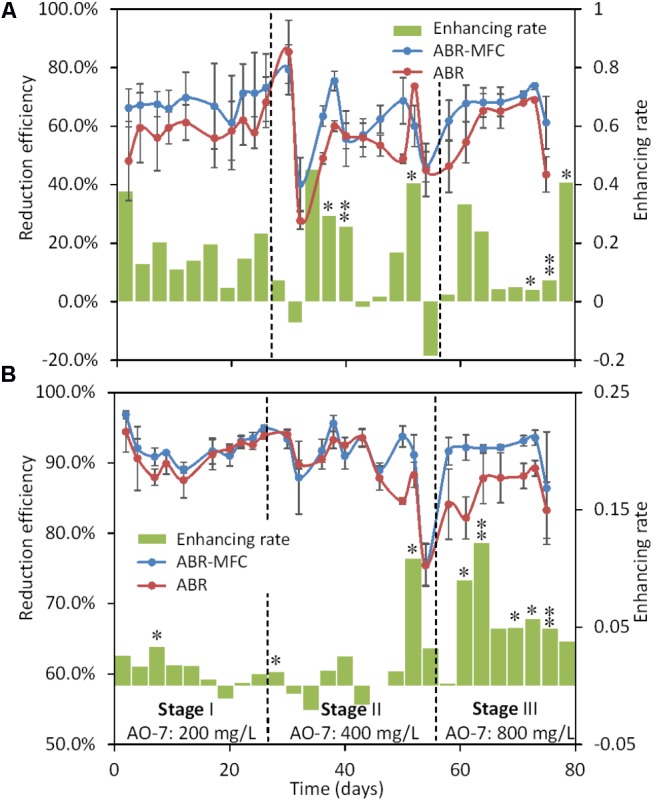
The enhancement of MFC-ABR on AO-7 reduction in chamber-1 **(A)** and the whole reactor **(B)**. (^∗^*p* < 0.05; ^∗∗^*p* < 0.01).

On average, 58.3 ± 7.9% and 65.5 ± 10.5% of AO-7 was reduced in the first chambers of ABRs and MFC-ABRs, respectively (**Figure 2A**), indicating that chamber-1 played a major role in AO-7 reduction. Furthermore, higher reduction efficiencies were detected in the first chambers of MFC-ABRs than those of ABRs over the experiments (excepted for day 15, 40, and 47). AO-7 showed no significant concentration difference (<2%) before and after it flow through the cathode zone (5 cm below cathode), suggesting that the AO-7 reduction was mainly occurred in the anodic sludge. In contrast to the inhibition of AO-7 on electricity generation (**Figure 1**), these results evidenced an enhancement of electricity generation on AO-7 reduction.

Regarding the whole reactor, ABR and MFC-ABR showed similarly AO-7 reduction efficiency (90.1 ± 2.1% vs. 91.4 ± 1.5%, respectively) in the first two stages (**Figure 2B**). In stage III, however, the average reduction efficiency in ABR was 85.2 ± 2.7%, significantly (*p* < 0.01) lower than the first two stages. In contrast, the AO-7 reduction efficiency in MFC-ABR was 91.6 ± 2.1% in stage III, comparable to the first two stages, indicating that MFC-ABR maintained a high treatment capacity even with high concentration of AO-7. Therefore, MFC-ABR would be particularly preferred in the treatment of wastewater containing high concentration of azo dyes. The azo dye reduction efficiency in MFC-ABR is comparable to several recently developed systems including ABR-biocatalyzed electrolysis system (ABR-BES), anaerobic sludge reactor-biocatalyzed electrolysis system (ASR-BES) and microbial fuel cell-aerobic reactors (MFC-AR) (Fernando et al., 2013; Cui et al., 2014; Kong et al., 2015; Thung et al., 2018). In contrast to the energy-consuming operations in those reactors (e.g., electrode polarization, aeration), net electricity generation can be obtained in MFC-ABR suggesting an advantage of MFC-ABR for application.

Similar to AO-7 reduction, COD removed mainly occurred in the first chamber in both MFC-ABR and ABR (**Supplementary Figure S2**). Higher COD removal was observed in MFC-ABR relative to ABR for all stages. The CE increased along the flow direction in MFC-ABR: chamber-1 (1.09 ± 0.22%) < chamber-2 (5.73 ± 1.64%) < chamber-3 (9.81 ± 1.19%) (**Supplementary Figure S3**). This was consistent with results that the first chamber removed most COD but generated lowest voltage. Moreover, the CE decreased with the increase of AO-7 concentration: stage-I (6.89 ± 0.23%) > stage-II (5.44 ± 0.95%) > stage-III (4.29 ± 0.67%), probably due to the increasing electron consumption by AO-7. The reported MFC CEs varied largely (from less than 1% to over 90%) (Kim et al., 2010, 2011; Zhang et al., 2017). The relative low CE in MFC-ABR indicated that most electrons were consumed by microbial growth, methanogenesis or the other electron acceptors (e.g., AO-7, oxygen, CO_2_) other than microbial anode reduction.

#### The Degradation Products and Toxicity Evaluation in ABR and MFC-ABR

**FIGURE 3:**
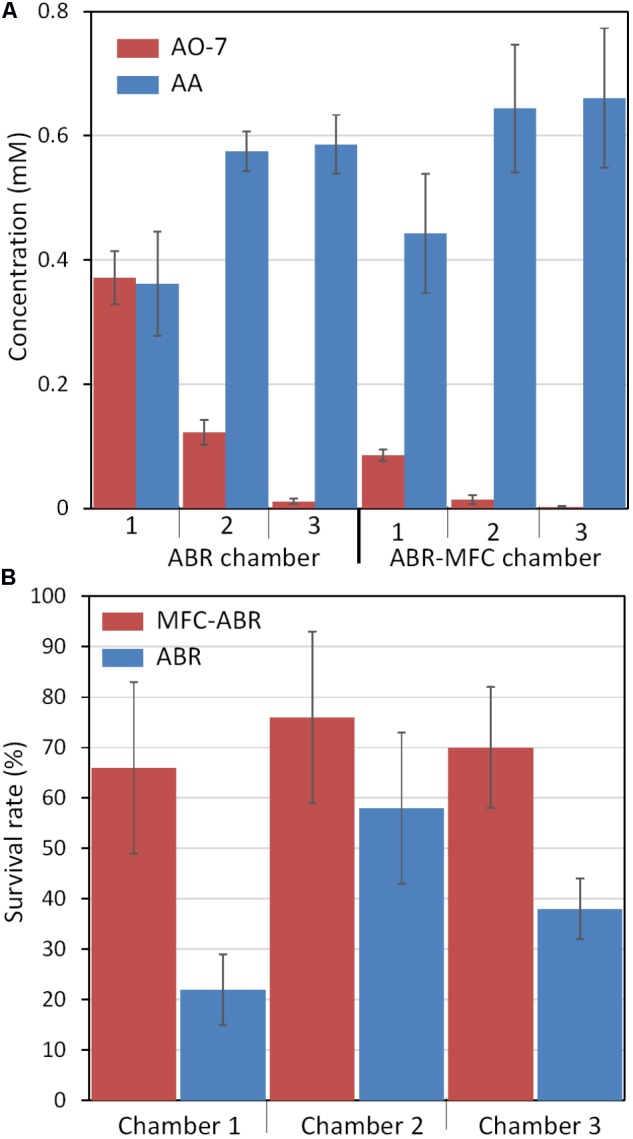
AO-7 reduction products **(A)** and toxicities **(B)** in ABRs and MFC-ABRs.

Microbial or electrochemical reduction of AO-7 generates 4-aminobenzenesulfonic acid (AA) and 1-amino-2-naphthol (AN) in anaerobic environments (Mu et al., 2009). LC-MS also showed a more rapid removal of AO-7 in each chamber of MFC-ABR than ABR (**Figure 3A**). AA was detected in all chambers while no AN was detected due to its unstable and auto-oxidizing property in low oxygen environments (**Figure 3A**) (Kong et al., 2015). Correspondingly, more AA was generated in MFC-ABR and accumulated along the flow direction. AA concentrations in ABR (0.58 ± 0.03 mM) or MFC-ABR (0.67 ± 0.11 mM) could not balance the removal of AO-7 (2.2 ± 0.1 mM) in both reactors, and the adsorption of AA to sludge has been demonstrated to be negligible (Kong et al., 2015), suggesting that most AA was further degraded in both ABR and MFC-ABR.

Detoxification was the main aim of wastewater treatment. However, toxicity has not been tested in previous BESs for treatment of azo dye-containing wastewaters. In this study, the survival rate of *Daphnia magna*, one of the most commonly used toxicity tests (Bozich et al., 2017), was used to evaluate the detoxification capacities of ABR and MFC-ABR (**Figure 3B**). The effluents of all chambers in MFC-ABR at stage III showed higher *Daphnia magna* survival rate than that of the ABR effluents, suggesting a lower toxicity of MFC-ABR effluents. Both ABR and MFC-ABR showed the highest toxicity in chamber-1 effluents, suggesting the necessity of the following two chambers in AO-7 detoxification (**Figure 3B**). Moreover, toxicity of the effluent was not proportional to the concentration either AO-7 or AA. It can be seen that toxicity is a combined effects of various chemical or biological components in the effluents rather than one or certain contaminants.

### Evaluation of the Microbial Activity and Sludge Granule Development

Both bio-reduction and electrochemical reduction may contribute to the AO-7 reduction in ABR and MFC-ABR. The anode redox potential in MFC-ABR maintained relatively stable at -0.25 ± 0.02 V (vs. SHE) while that of the anode (in open-circuit) in ABR was -0.3 ± 0.02 V (vs. SHE). Mu et al. (2009) have reported that AO-7 can be slowly reduced by a graphite electrode at -0.29 V (vs. SHE) and the reduction efficiency increased at lower electron potential, indicating that more electrochemical AO-7 reduction occurred in ABR due to the lower anode potential. Therefore, the higher total AO-7 reduction efficiency in MFC-ABR was mainly caused by bio-reduction rather than electrochemical reduction.

**FIGURE 4:**
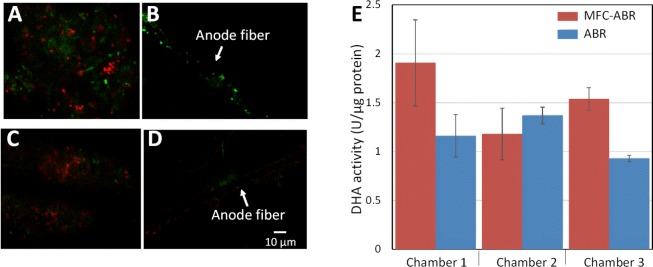
Microbial and DHA activity in chamber 3 of ABR and MFC-ABR. **(A,C)** Microbial activity of the sludge in MFC-ABR and ABR, respectively; **(B,D)** microbial activity of the biofilms on carbon fires of MFC-ABR and ABR, respectively. **(E)** DHA activity of the sludge in different chambers of MFC-ABR and ABR. Green color in **(A–D)** indicates microbial cells have high metabolism activity and intact cell membrane while red color indicates microbial cells with low metabolism activity and damaged membrane. Scale bar indicates 10 μm.

Biomass and bioactivity of the sludge play a key role in contaminant removal in wastewater treatment. Protein-based biomass assessment showed no significant difference among the chambers of ABR and MFC-ABR (**Supplementary Figure S4**). However, microbial cells in the sludge showed obviously higher viability in MFC-ABR (RV = 0.64 ± 0.11) than that in ABR (RV = 0.47 ± 0.08, **Figure 4**). Single layer biofilms (0 – 3 μm in thickness) were observed on both MFC-ABR and ABR anode fibers, and possible impair on biofilm structure could not be ruled out when the anodes were sampled from sludge. MFC-ABR anode biofilms also showed significantly higher viability (RV = 0.91 ± 0.07) than that of the ABR biofilms (RV = 0.44 ± 0.05). This is consistent to our previous results that electrode could serve as favorable electron acceptor and stimulate the metabolism activity of microbial cells under anaerobic condition (Yang et al., 2015). To further confirm the higher respiratory activity in MFC-ABR, the activity of DHA, an essential component in bacterial electron transfer (Stolz, 2001; Shi et al., 2009), was measured. DHA activities in chamber-1 and chamber-3 of MFC-ABR were significantly higher than that of ABR (**Figure 4**), indicating a higher electron transfer activity in these cambers. It should be noted that DHA is a group of intracellular redox enzymes. Higher DHA activity accounts for higher intracellular electron transfer activity (e.g., AO-7 reduction) but cannot ensure a higher EET activity, because all microbes have DHA but only a minor portion of microbes have EET capability (Koch and Harnisch, 2016).

The development of sludge granule is also an important factor affecting the treatment capacity in the reactors. The numbers of big sludge granules (diameter ≥ 100 μm) in all chambers of MFC-ABR were significantly higher than that in ABR (**Supplementary Figure S5**). Sludge granules with a diameter over 150 μm were detected in all MFC-ABR chambers but not in ABR. The results suggested that the growth of sludge particles was also improved by anode respiration which might contribute to higher AO-7 removal efficiency in MFC-ABR.

### Microbial Community Structure and Function

#### The Changes of Microbial Community Structure

A total of 261, 162 effective reads were retrieved after trimming the adapters, barcodes, primers and filtering the low quality reads including chimeras. The number of OTUs at a cutoff level of 3% ranged from 1624 to 1983 among different samples with slightly higher numbers observed in ABR (1859 ± 115) than in MFC-ABR (1775 ± 88). Accordingly, the alpha diversity estimated by the Chao1 and Shannon indexes in MFC-ABR was significantly lower than ABR (*p* < 0.05). The first MFC-ABR chamber showed significantly higher diversity than the following two chambers while no difference was observed among ABR chambers (**Supplementary Table S1**). Principal Coordinate Analysis (PCoA) based on Bray-Curtis distance of OTUs showed that the samples could be grouped into MFC-ABR and ABR clustered by PCoA1 axes (**Supplementary Figure S6**). The above results indicated a significant role of electricity generation in shaping microbial community structure. RDA was used to evaluate the associations between reactor variables and the communities. The first two axes explained 66.3 and 45.8% of the microbial community variations in MFC-ABR and ABR, respectively. DO in MFC-ABR (*r*^2^ = 0.66, *p* = 0.04) while ORP (*r*^2^ = 0.5466, *p* = 0.07) and removal efficiency (*r*^2^ = 0.64, *p* = 0.03) in ABR were the major drivers affecting community structure and compositions.

With RDP Classifier at an 80% threshold, all effective microbial sequences were assigned to different taxa levels. Among a total of 28 detected phyla (**Supplementary Figure S7**), *Proteobacteria* was the most abundant phylum that accounted for 43.7 ± 7.3 % and 33.7 ± 7.4% of total microbial sequences in MFC-ABR and ABR, respectively. *Firmicutes* (15.7 ± 4.0% and 18.5 ± 3.4%), *Bacteroidetes* (8.0 ± 0.7% and 7.8 ± 1.7%), *Chloroflexi* (10.0 ± 3.1% and 12.1 ± 3.3%), *Actinobacteria* (2.5 ± 0.5% and 3.1 ± 0.5%) also dominated communities in both ABR and MFC-ABR. Within phylum *Proteobacteria*, *γ-proteobacteria* (21.1 ± 4.3%) and *β-proteobacteria* (8.2 ± 2.6%) had significantly higher abundances in MFC-ABR than ABR (14.3 ± 6.1%, 4.7 ± 1.2%).

At the genus level, *Pseudomonas* (6.7 – 9.9%), *Sporolactobacillus* (3.2 – 4.7%), *Clostridium sensu stricto* (1.7 – 3.9%), *Lewinella* (1.5 – 1.9%) and *Longilinea* (1.1 – 1.6%) showed >1% abundance in all MFC-ABR chambers, representing the core consortia in MFC-ABR. Compared to the ABR community, the abundances of seven genera were higher whereas nine genera were lower than those in the MFC-ABR (*P* < 0.05, **Table 1**). Among genera enriched in the MFC-ABR, *Pseudomonas* showed increasing abundance along the flow direction, which was consistent with its versatile capacities including azo dye reduction, electricity generation, aromatic compounds biodegradation and fermentation (Galai et al., 2015). The other MFC-ABR-enriched genera have also shown capacities such as EET (*Serratia*, *Stenotrophomonas*), azo-dye reduction (*Stenotrophomonas*) or aromatic compounds degradation (*Dysgonomonas*, *Brevundimonas*, and *Achromobacter*) (Jin et al., 2015; Paul et al., 2015; Hao et al., 2016), and most of them showed increasing abundances along with the flow direction. On the other hand, the abundances of genera associated with fermentation and methanogenesis were lower in MFC-ABR relative to ABR, such as *Acinetobacter*, *Cloacibacillus* and *Methanobacterium* (Krivushin et al., 2010; Lovley, 2011; Looft et al., 2013).

**Table 1:** Genera (abundance > 0.1%) with significantly different abundances between MFC-ABR and ABR.

	*T*-test		ANOVA		Welch test		Kruskal–Wallis	
Genus	*t*^a^	*p*	*F*	*p*	*F*	*p*	χ^2^	*p*
*Pseudomonas*	2.39	0.0298	5.69	0.0298	5.69	0.0337	6.11	0.0134
*Serratia*	2.49	0.0370	6.22	0.0239	6.22	0.0370	12.79	0.0003
*Dysgonomonas*	3.14	0.1152	9.86	0.0063	9.86	0.0115	4.87	0.0269
*Stenotrophomonas*	3.32	0.0043	11.03	0.0043	11.03	0.0080	9.55	0.0016
*Brevundimonas*	3.58	0.0066	12.84	0.0025	12.84	0.0066	8.75	0.0030
*Achromobacter*	3.69	0.0046	13.60	0.0020	13.60	0.0046	6.11	0.0129
*Shinella*	–5.09	0.0003	25.91	0.0001	25.91	0.0003	10.67	0.0010
*Cloacibacillus*	–4.47	0.0004	19.97	0.0004	19.97	0.0007	9.01	0.0022
*Syntrophomonas*	–3.55	0.0027	12.60	0.0027	12.60	0.0048	7.98	0.0029
*Methanobacterium*	–3.53	0.0074	12.46	0.0028	12.46	0.0074	9.01	0.0026
*Acinetobacter*	–2.86	0.0114	8.18	0.0114	8.18	0.0123	5.90	0.0151
*Syntrophobacter*	–2.78	0.0182	7.75	0.0133	7.75	0.0182	5.68	0.0140
*Zavarzinella*	–2.68	0.0164	7.19	0.0164	7.19	0.0176	4.87	0.0226
*Armatimonadetes_gp5*	–2.56	0.0209	6.56	0.0209	6.56	0.0211	4.49	0.0311
*Tenacibaculum*	–2.46	0.0258	6.04	0.0258	6.04	0.0275	4.31	0.0365
*Clostridium sensu stricto*	–2.39	0.0297	5.70	0.0298	5.60	0.0298	4.68	0.0303
*Gemmata*	–2.33	0.0330	5.45	0.0330	5.45	0.0359	3.95	0.0350


*^a^Negative values mean lower abundance in MFC-ABR than ABR.*

#### The Prediction of Microbial Community Function

**FIGURE 5:**
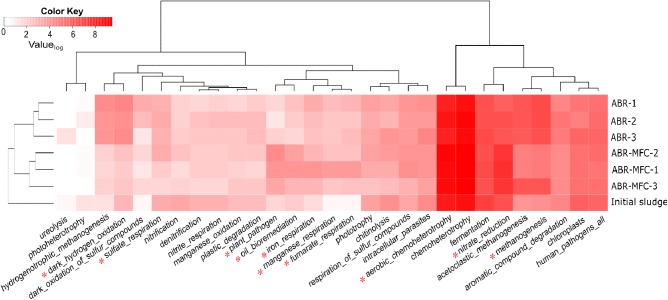
Heat-map and cluster profiles of microbial functions in different chambers of ABR and MFC-ABR. Color-key indicates the Log_2_-value of the average OTU number (*n* = 3). Only the functions with significant OTUs (i.e., average OUT number in all samples > 1) were shown. ^∗^Indicates functions with significantly different OTU numbers in ABR and MFC-ABR.

The FAPROTAX database has been proposed as a tool to predict possible environmental functions of microbial communities (Louca et al., 2016). FAPROTAX predicted 92 functions of OTUs in the sludge samples and 30 (with average OTU > 1) of them were shown in **Figure 5**. The functional compositions of ABR chambers and MFC-ABR chambers were well separated, indicating functional diversification in the two types of reactor. Chemoheterotrophy (34.9 ± 1.7%) was the predominant process predicted by FAPROTAX in both reactors, followed by nitrate reduction (7.4 ± 2.3%) and fermentation (6.6 ± 1.6%). Ten functions showed significantly different OTU abundances between ABR and MFC-ABR. Seven of them, including oil bioremediation, aerobic chemoheterotrophy, plant pathogen, iron respiration, manganese respiration, fumarate respiration and nitrate respiration were enhanced in MFC-ABR relative to ABR while the other three (hydrogen-oxidization, sulfate respiration and methanogenesis) were suppressed. Consistent with RDA and community analyses, the chamber-specific functional structure also suggested a sequentially segregated biochemical processes in both ABR and MFC-ABR. Compared with the enhanced nitrate respiration, sulfate respiration was gradually decreased from chamber 1–3 in MFC-ABR, indicating that the electricity generation suppressed sulfate respiration but co-existed with nitrate respiration (**Figure 5**). Moreover, the abundances of most electricity-stimulated respirations including iron-, manganese-, fumarate- and nitrate-respirations showed the highest abundances in the first chamber and decreased along the flow direction, suggesting that those functions were not only enhanced by electricity generation but also by azo reduction. The profiles of those anaerobic respirations coincided with the higher microbial viability and DHA activity shown in **Figure 4**, as the DHA and *c*-type cytochromes were commonly shared as key electron transfer components in those respirations (Shi et al., 2009). Anaerobic respirations (except for sulfate reduction) and oil-bioremediation, mainly aromatics degradation, always showed consistent abundances in both ABR and MFC-ABR, indicating a synergistic relationship among the bacterial respiration and aromatics biodegradation under anaerobic conditions.

**FIGURE 6:**
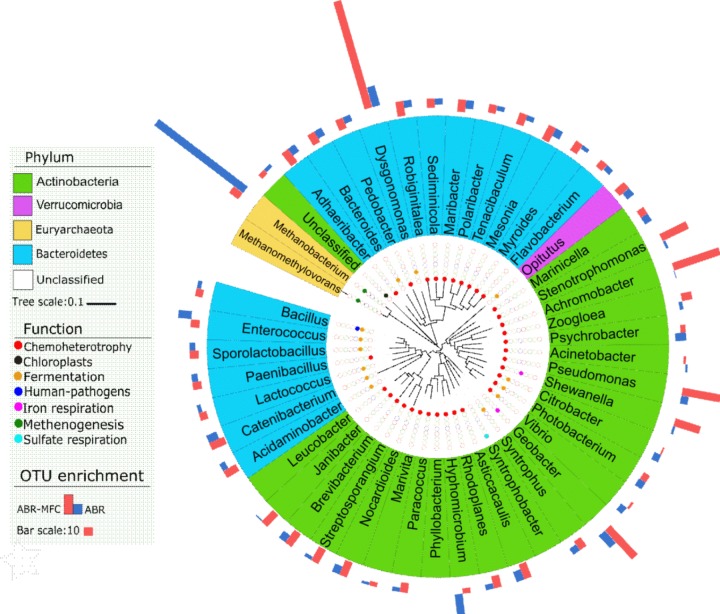
The genera with significantly different (*p* < 0.05, *n* = 3) OTU numbers among ABR, MFC-ABR and initial sludge and their annotated functions in FAPROTAX. The background color of each genus indicate its phylum. The circles filled with different colors indicate the functions of the corresponding genus. The bars in the outmost layer indicate the enhanced-fold of the genus in MFC-ABR and ABR, relative to the genus in initial sludge.

To identify the key bacteria contributing to the functional differences in **Figure 5**, FAPROTAX identified 48 genera that shared by ABR and MFC-ABR but differ in their relative abundances (*p* < 0.05) (**Figure 6**). Those genera involved in eight functions, including iron respiration (2 genera), fermentation (13 genera), methenogenesis (2 genera), chemoheterotrophy (32 genera), human pathogens (1 genus), sulfate respiration (1 genus), photosynthesis (1 genus). The enhancements of *Pseudomonas, Stenotrophomonas, Achromobacter, Dysgonomonas* in MFC-ABR were also noted by FAPROTAX. Two typical electricity generating bacteria, *Shewanella* and *Geobacter* (Shi et al., 2009), were predicted to play key roles in the enhancement of iron respiration in MFC-ABR. In contrast, 11 fermentation bacteria and two methenogenesis archaea (*Methanobacterium* and *Methanomethylovorans*) were the key members contributing to the enrichment of fermentation and methenogenesis in ABR. It should be noted that the other significantly differed functions between ABR and MFC-ABR were caused by bacteria without significant abundances or not included in FAPROTAX.

Community composition and functional predication suggested that the low energy-generating processes such as fermentation, methenogenesis and sulfate reduction were suppressed by electricity generation due to the relative high redox potential of MFC anode. Previous transcriptomic analyses have showed that electron acceptor redox potential could alter bacterial metabolism pathway (Lian et al., 2016). Our Geochip data of sediment MFC showed that anodes could enhance the functional genes such as nitrate reductase, reductive dehalogenase, aromatic degradation genes, partially supporting the community composition profiles in this study (Yang et al., 2013). The co-enriched versatile bacteria for respiration and biodegradation by microbial anode reduction might also contribute to the higher AO-7 degradation and detoxification in MFC-ABR. However, it should be noted that FAPROTAX prediction only provided possible microbial functions, further evidences such as gene-expression, comprehensive chemical analyses are needed to test the reliability of the prediction.

## Conclusion

Microbial anode respiration could significantly enhanced AO-7 degradation and detoxication in MFC-ABR compared with ABR, especially under high AO-7 concentration, which could be attributed to the higher microbial activity, dehydrogenase activity and larger sludge granule size in MFC-ABR. Moreover, microbial anode respiration dramatically reshaped the community structure and decreased the diversity of the microbial communities. *Pseudomonas* was the most abundant genus enriched by electricity generation. In contrast to the suppressed bacteria capable of methanogenesis and sulfate respiration, the co-enriched bacteria with versatile respiration (e.g., EET, fumarate and nitrate respiration) and biodegradation capabilities provided useful information to understand the enhanced degradation and detoxication of AO-7 by microbial anode respiration.

## Author Contributions

YY and MX designed the study. YY and OL operated the experiments. YY, OL, MX, GK, BW, XL, EL, and JL analyzed and discussed the results. YY, OL, and FL wrote the paper. All the authors agree to be accountable for the content of the work.

## Conflict of Interest Statement

The authors declare that the research was conducted in the absence of any commercial or financial relationships that could be construed as a potential conflict of interest.
