# Supplementary material for: Deciphering the Anode-Enhanced Azo Dye Degradation in Anaerobic Baffled Reactors Integrating With Microbial Fuel Cells
Source: Front Microbiol. 2018 Sep 6;9:2117. doi: 10.3389/fmicb.2018.02117 (PMC6135904; doi:10.3389/fmicb.2018.02117)
Supplement: Supplementary file 1 [file Table_1.DOCX]

Supplementary Material

**Deciphering the anode-enhanced azo dye degradation in anaerobic baffled reactors integrating with microbial fuel cells**

Yonggang Yang^1,2^, Ou Luo^1^, Guannan Kong^1^, Bin Wang^1^, Xiaojing Li^1^, Enze Li^1^, Jianjun Li^1,2^, Feifei Liu^1^, Meiying Xu^1,2^ *

^1^ Guangdong Provincial Key Laboratory of Microbial Culture Collection and Application, Guangdong Institute of Microbiology, 510070, Guangzhou, China, ^2^ State Key Laboratory of Applied Microbiology Southern China, 510070, Guangzhou, China

* Correspondence:

Dr. Meiying Xu

xumy@gdim.cn (M Xu)

**Supplementary Tables and Figures**

Table S1 Alpha diversity of the communities in ABR and MFC-ABR

| ID | resample | Richness | ACE | Chao1 | Simpson | Shannon | PD |
| --- | --- | --- | --- | --- | --- | --- | --- |
| MFC-ABR1-chamber1 | 3036 | 2082 | 19618 | 16904 | 0.990 | 9.5 | 210 |
| MFC-ABR2- chamber 1 | 3036 | 2134 | 19442 | 16905 | 0.992 | 9.7 | 219 |
| MFC-ABR3- chamber 1 | 3036 | 2120 | 19458 | 15891 | 0.992 | 9.5 | 207 |
| MFC-ABR1- chamber 2 | 3036 | 1897 | 16007 | 14057 | 0.991 | 9.3 | 200 |
| MFC-ABR2- chamber 2 | 3036 | 2084 | 16798 | 14490 | 0.981 | 9.0 | 198 |
| MFC-ABR3- chamber 2 | 3036 | 2189 | 19147 | 16000 | 0.990 | 9.5 | 206 |
| MFC-ABR1- chamber 3 | 3036 | 1999 | 15970 | 13632 | 0.986 | 9.1 | 199 |
| MFC-ABR2- chamber 3 | 3036 | 2183 | 19649 | 16723 | 0.989 | 9.4 | 204 |
| MFC-ABR3- chamber 3 | 3036 | 1845 | 20689 | 18341 | 0.989 | 9.6 | 207 |
| ABR1- chamber 1 | 3036 | 2155 | 22504 | 18759 | 0.994 | 9.8 | 222 |
| ABR2- chamber 1 | 3036 | 2047 | 18507 | 17098 | 0.993 | 9.6 | 212 |
| ABR3- chamber 1 | 3036 | 1981 | 20342 | 16255 | 0.994 | 9.9 | 231 |
| ABR1- chamber 2 | 3036 | 2085 | 19651 | 17434 | 0.994 | 9.8 | 225 |
| ABR2- chamber 2 | 3036 | 1876 | 14135 | 12820 | 0.981 | 9.0 | 194 |
| ABR3- chamber 2 | 3036 | 2072 | 23644 | 19933 | 0.994 | 9.9 | 231 |
| ABR1- chamber 3 | 3036 | 2296 | 16273 | 14691 | 0.991 | 9.4 | 202 |
| ABR2- chamber 3 | 3036 | 2012 | 18773 | 17987 | 0.994 | 9.8 | 226 |
| ABR3- chamber 3 | 3036 | 1824 | 21601 | 17032 | 0.993 | 9.7 | 229 |


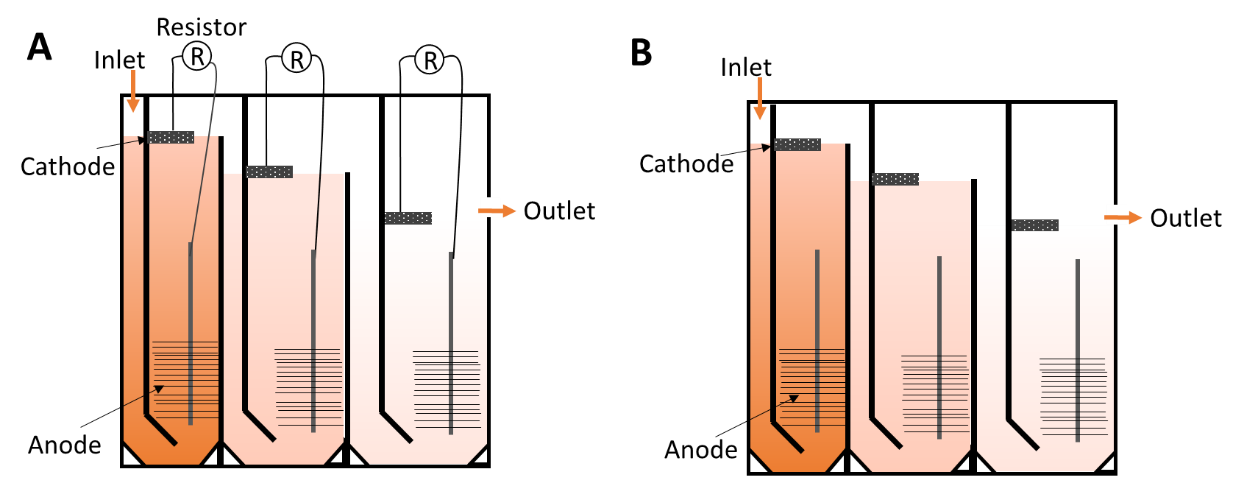


Fig. S1 Schematic of the MFC-ABR (A) and ABR (B) reactors.


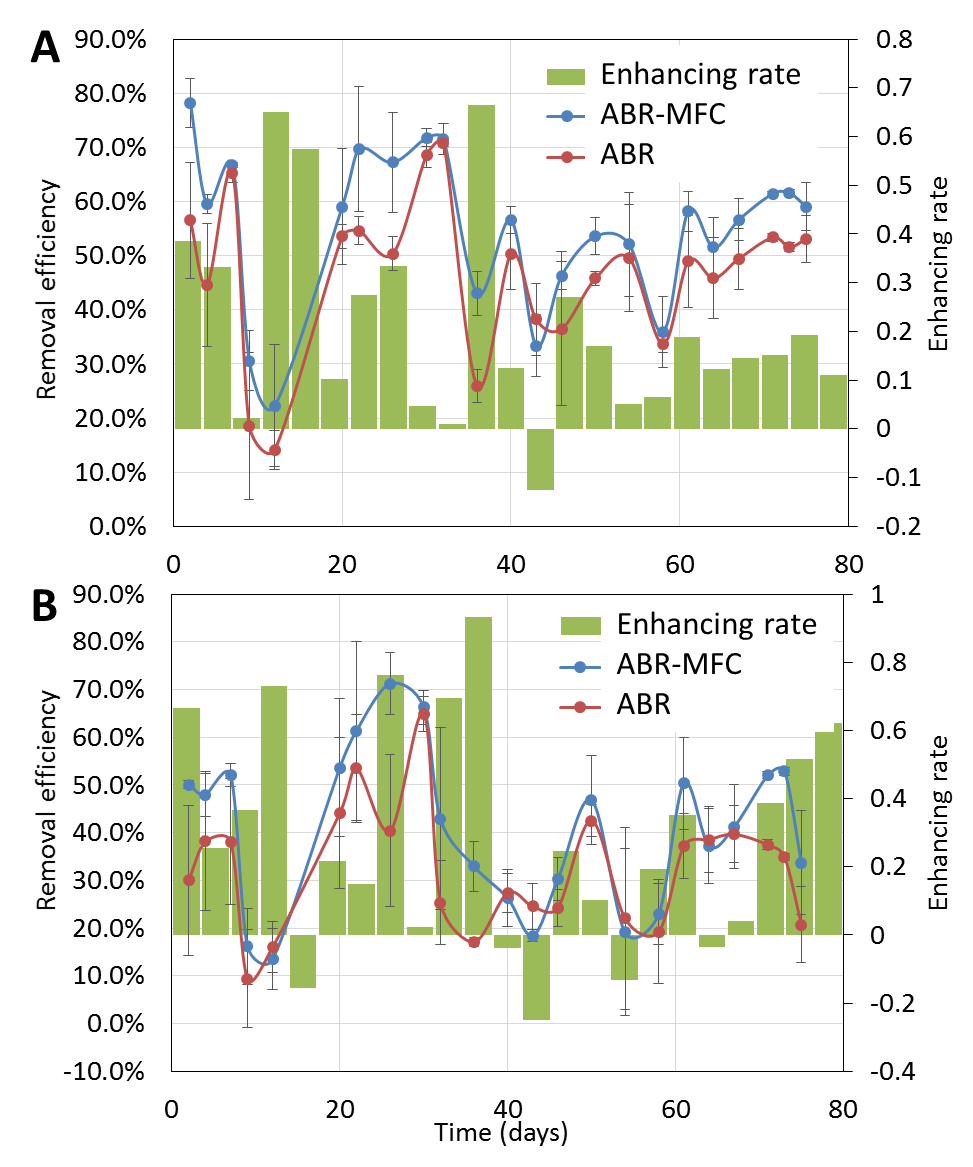


Fig. S2 A comparison of the COD removal in the ABR and MFC-ABR. A, the total removal efficiency in the two reactors; B, the removal efficiency in the first chambers ABR and MFC-ABR. Green bars indicates the enhancing-rate of MFC-ABR relative to ABR at different sampling times.


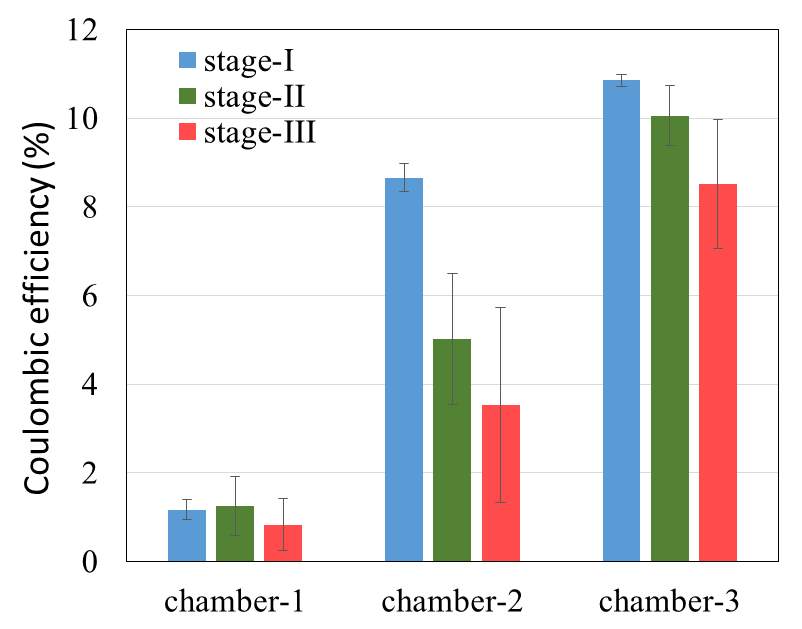


Fig. S3 The Coulombic efficiency in different reactors and stages.


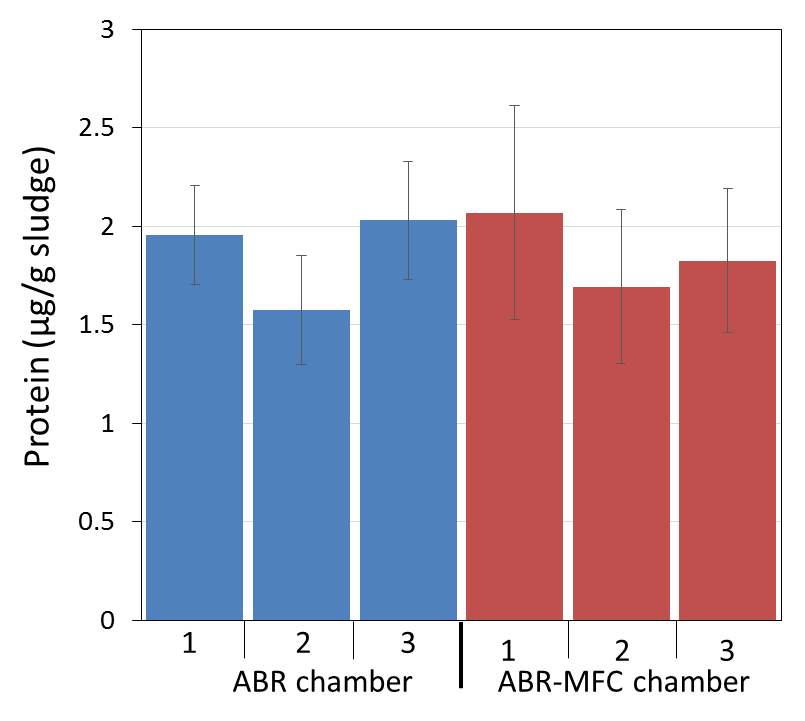


Fig. S4 Bioamss in the sludge of ABR and MFC-ABR


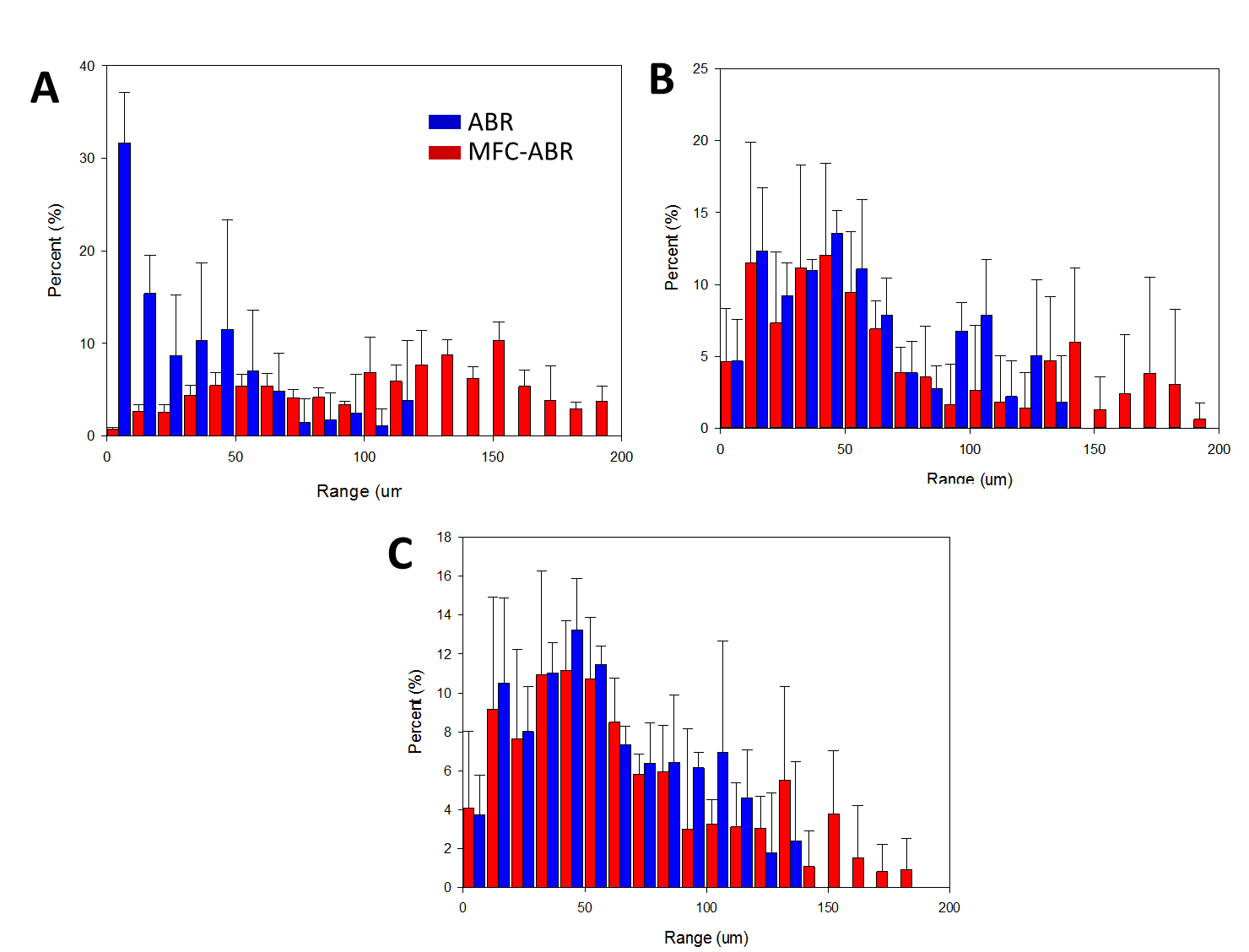


Fig. S5 Particle size of the sludge in chamber 1 (A), chamber 2 (B) and chamber 3 (C) of ABR and MFC-ABR.


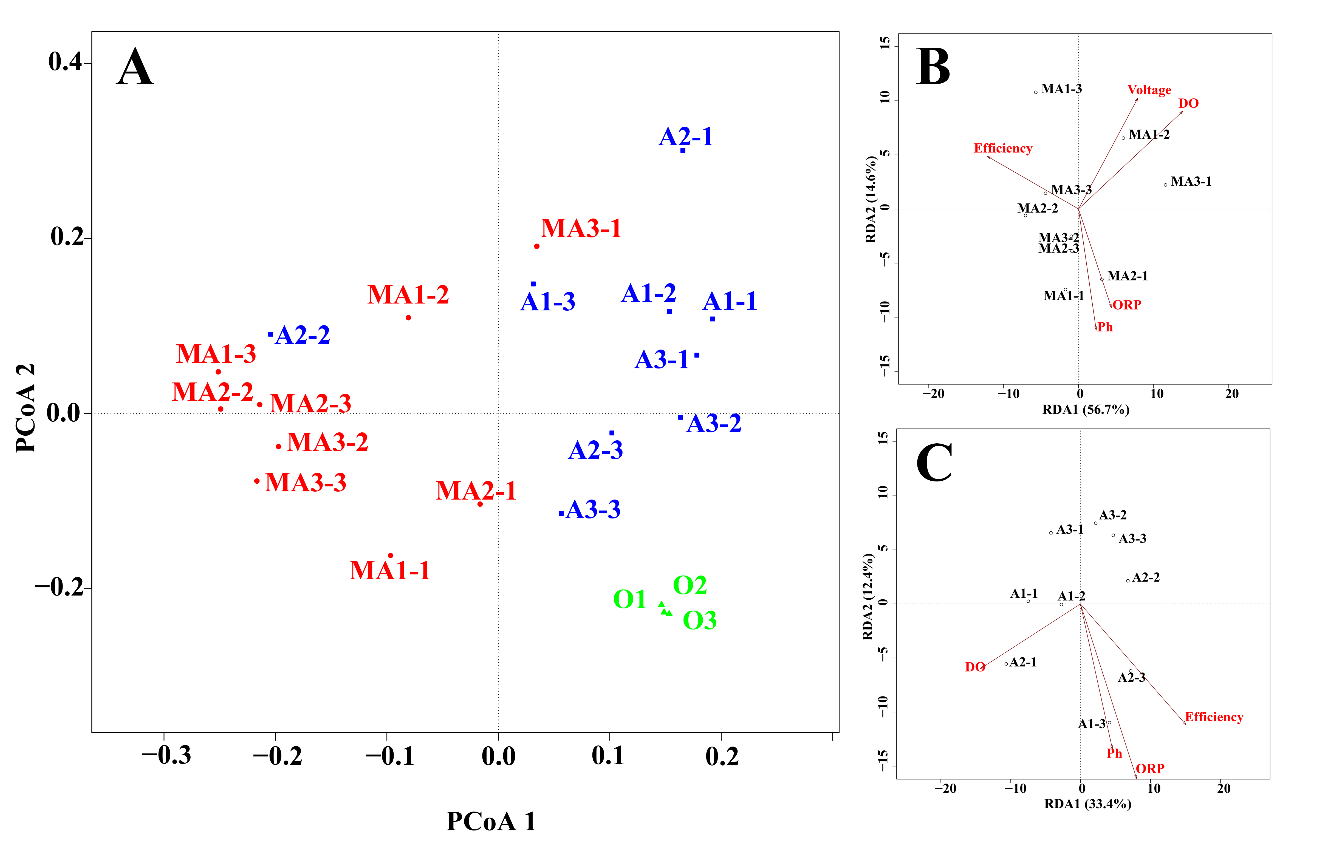


Fig. S6 PCoA cluster of MFC-ABR and ABR communities (A) and RDA clusters of MFC-ABR communities (B) and ABR communities (C). The blue dots A1, A2 and A3 LO7-9 indicate the triplicated ABR; the red dots MA1, MA2 and MA3 indicate the triplicated MFC-ABR; the number after the dash indicate the chamber order; and the green dots indicates the original sludge samples. Efficiency indicate the AO-7 removal efficiency and voltage indicate the voltages generated in different MFC-ABR chambers.


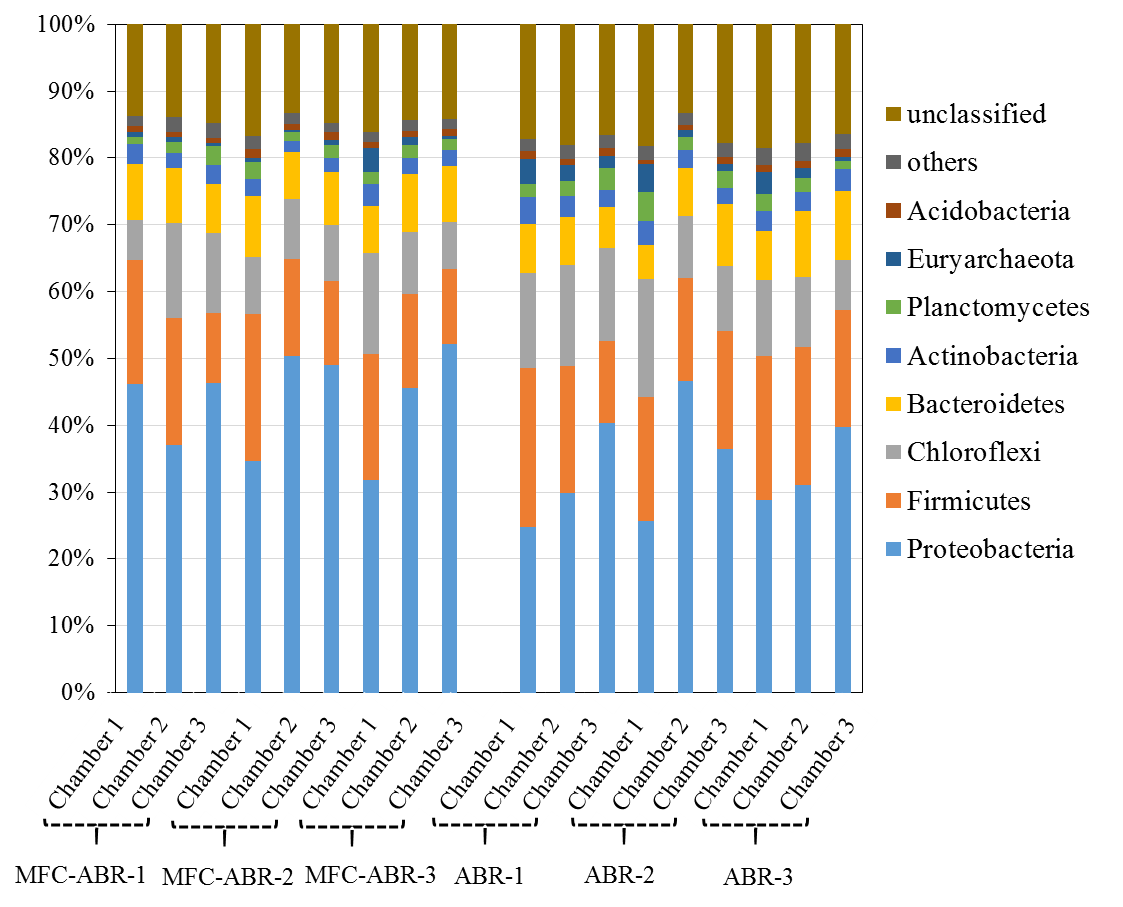


Fig. S7 phyla composition of each chamber in MFC-ABR and ABR.
